# Evidence-based Management Strategies for Treatment of Chronic Wounds

**Published:** 2009-06-04

**Authors:** Frank Werdin, Mayer Tennenhaus, Hans-Eberhardt Schaller, Hans-Oliver Rennekampff

**Affiliations:** ^a^ BG-Trauma Center, Department of Plastic, Hand and Reconstructive Surgery, Burn Centre, University of Tübingen, Germany; ^b^ Division of Plastic Surgery, UCSD School of Medicine, San Diego, CA; ^c^ Department of Plastic, Hand and Reconstructive Surgery, Medical School Hannover, Germany

## Abstract

The care and management of patients with chronic wounds and their far-reaching effects challenge both the patient and the practitioner. Further complicating this situation is the paucity of evidence-based treatment strategies for chronic wound care. After searching both MEDLINE and Cochrane databases, we reviewed currently available articles concerning chronic wound care. Utilizing this information, we have outlined a review of current, evidence-based concepts as they pertain to the treatment of chronic wounds, focusing on fundamental treatment principles for the management of venous, arterial, diabetic, and pressure ulcers. Individualized treatment options as well as general wound management principles applicable to all varieties of chronic wounds are described. Classification and treatment guidelines as well as the adoption of the TIME acronym facilitate an organized conceptional approach to wound care. In so doing, individual aspects of generalized wound care such as debridement, infection, and moisture control as well as attention to the qualities of the wound edge are comprehensively evaluated, communicated, and addressed. Effective adjuvant agents for the therapy of chronic wounds including nutritional and social support measures are listed, as is a brief review of strategies helpful for preventing recurrence. An appreciation of evidence-based treatment pathways and an understanding of the pathophysiology of chronic wounds are important elements in the management of patients with chronic wounds. To achieve effective and long-lasting results, a multidisciplinary approach to patient care, focused on the education and coordination of patient, family as well as medical and support staff can prove invaluable.

The treatment and care of chronic wounds may be an unglamorous aspect of medical practice, but for both the patient and the society, the resulting morbidity and cost are considerable. Indeed much of the medical establishment, whether through lack of confidence, training, interest, or remunerative potential, continues to perceive this to be under the province of someone else.

With the population advancing in age, increasing in weight and with the resultant comorbidities of diabetes and venous insufficiency, an increase in the number of patients with chronic wounds has been reported.^1^–^3^ It has been estimated that approximately 1% of the population will develop leg ulceration in the course of their lifetime. In the United States alone, chronic wounds affect 3 million to 6 million patients and treating these wounds costs an estimated $5 billion to $10 billion each year. Of particular concern, we and others have noted an increase in the number of patients who have been insufficiently treated for their chronic wounds over protracted courses.^2^ We believe that established treatment pathways for chronic wounds can prove highly relevant in daily practice and as a result we have outlined current concepts concerning the treatment of chronic wounds, focusing on fundamental treatment principles for the management of venous, arterial, diabetic, and pressure ulcers.

## DEFINITION AND PATHOLOGY

**Figure 1:**
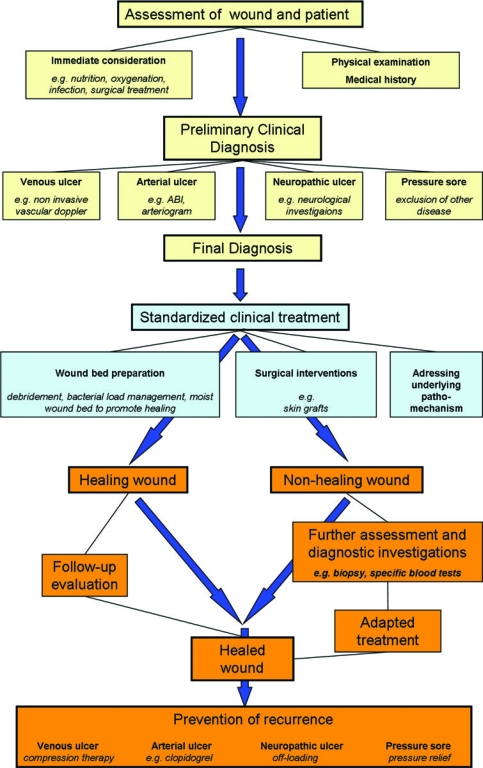
A management strategy for treatment of chronic wounds.

Chronic wounds are defined as wounds, which have failed to proceed through an orderly and timely reparative process to produce anatomic and functional integrity over a period of 3 months.^4^ All wound types have the potential to become chronic and, as such, chronic wounds are traditionally divided etiologically. Identifying and treating the underlying aetiology of a chronic wound such as venous insufficiency, arterial perfusion, diabetes, or unrelieved pressure as well as systemic factors such as nutritional status, immunosuppression, and infection that may contribute to poor wound healing are key to successful wound treatment.^4^ General treatment principles for the management of chronic wounds are demonstrated in Figure 1. The most commonly encountered chronic wound is the lower extremity ulcer; these are generally vascular or diabetic in nature and account for up to 98% of all lower extremity wounds.^5^

Chronic wounds are often identified by the presence of a raised, hyperproliferative, yet nonadvancing wound margin. Fibroblasts derived from the wound bed of chronic wounds of various etiologies represent a senescent, premature, or differentiated phenotype, which respond inefficiently to normal stimulatory messages.^4^,^6^,^7^ The local wound environment, rich in inflammatory products, and proinflammatory cytokines manifest an imbalanced enzymatic milieu consisting of an excess of matrix metalloproteases and a reduction in their inhibitors resulting in the destruction of the extra cellular matrix.^6^ The resultant profound inflammatory state is thought to be a significant factor influencing and delaying healing. Chronic inflammation, a hallmark of the nonhealing wound, may ultimately predispose these wound sites to potential malignant change. A detailed understanding of the mechanisms controlling the inflammatory response, tissue repair, and directed healing outcome is necessary for effective therapy of pathological tissue repair.

Correctly identifying the etiology of a chronic wound as well as the local and systemic factors that may be contributing to poor wound healing is key to successful wound treatment.^4^–^6^ In general, local tissue hypoxia with repetitive ischemia-reperfusion injury is considered a common pathogenesis in chronic wound development.^6^

## DIABETIC ULCER

Diabetic wounds and their pattern of chronicity appear to be multifactorial in nature. Once thought to be predominantly a disease of small vessels, large vessel contribution is increasingly recognized.^8^ Neuropathic diabetic ulcers require therapeutic regimens directed at several causative elements including the neuropathy, blood sugar control, revascularization as well as prevention strategies.^4^ The diagnosis of a diabetic neuropathy remains particularly challenging and is confirmed by history, clinical examination, and Semmes-Weinstein filament testing.^9^ Improving vascular flow, medical therapy for neuropathy, and surgical decompressions have all shown to contribute to effective management.^4^,^8^ Judicious diabetic control is critical and as with all chronic wounds, prevention, education, and examination are of paramount importance.^8^ All patients with pressure-induced, neuropathic diabetic foot wounds should receive an orthopedic evaluation for maximal pressure off-loading.^4^–^8^,^10^ Methods of offloading include crutches, walkers, wheelchairs, and a variety of protective and stabilizing footwear. Achilles tendon lengthening procedures, for example, have been shown to improve the rate of healing of neuropathic forefoot plantar ulceration by improving kinematics and reducing focal pressure effects.^4^ In addition, the transplantation of healthy living skin equivalents, cells that assist in ulcer healing by stimulating the release of growth factors and cytokines, has shown varying degrees of benefit in healing diabetic ulcers after judicious wound bed preparation.^4^

## VASCULAR ULCER

All patients with lower extremity ulcers should be assessed for arterial disease. Vascular ulcers, despite their characteristic location and appearance, merit a clinical vascular examination to identify and characterize the ulcer, distinguishing arterial from venous contributions.^4^,^11^ A relevant history and accurate clinical examination including assessment of cutaneous changes, dependent rubor, capillary refill, and claudication should be performed. The next diagnostic steps would generally be an assessment of the ABI (ankle/brachial index) as well as transcutaneous oximetry. The screening value for arterial disease is defined by a resting ABI ≤ 0.9. Transcutaneous oxygen tension (TcPo_2_) is thought to be a more effective marker of disease than Doppler assessment or ABI. A value less than 40 mm Hg is associated with impaired healing.^4^,^11^ TcPo_2_ levels are often helpful in predicting healing after amputation as well as assessing the success of vascular intervention. If an otherwise healthy patient presents with strong palpable dorsalis pedis and posterior tibialis pulses, no immediate further referral is generally required. A suspicion of arterial disease in the context of a patient with lower extremity ulcer should prompt referral to a vascular specialist (eg, vascular surgeon, angiologist).^4^,^11^

In cases of arterial ulcers, restoration of blood flow by revascularization is the intervention that will most likely lead to healing. Prior to surgery, an anatomic road map should be obtained by angiogram, duplex angiography, magnetic resonance angiography, computed tomography angiography, or contrast tomography angiography. The success of vascular intervention is confirmed by manifest pulses in the foot, improved ABI, or improved wound healing. In some patients, primary amputation must be considered, while in others limb preservation may be of utmost importance. The role of amputation in the management of complex extremity wounds needs to be considered in a complex risk-benefit analysis and carefully discussed with the patient.^4^,^11^

In cases of venous ulceration, gross arterial disease should be ruled out as described above and the specific venous etiology of the ulcer confirmed by color duplex scan.^4^ It is important to identify and distinguish deep and superficial system patency and competency. Venous ulcer healing rates improve when adequate compression therapy–specifically a class 3 high-compression system–is consistently applied. It is important to note, however, that compression therapy is contraindicated in cases of significant arterial insufficiency.^4^,^12^ If there is no evidence of significant deep system venous disease, venous hypertension can effectively be reduced by SEPS (subfascial endoscopic perforator surgery).^12^ Contemporary trends are toward less invasive vascular surgery interventions such as SEPS, superficial venous ablation, sclerotherapy, endovenous laser ablation, or valvuloplasty combined with compression therapy. These modalities have proven quite beneficial in improving rates of ulcer healing as well as decreasing ulcer recurrence, but only in combination with compression therapy.^12^

## PRESSURE ULCER

In patients with pressure ulcers or for those patients who are at high risk of developing pressure ulcers, a relevant history of mobility, previous immobility, neurological impairment (eg, paraplegia, multiple sclerosis), and a clinical assessment defining significant pressure points encountered in daily life are critical for establishing preventive and therapeutic interventions.^5^,^13^ Patient positioning and methods to reduce pressure-related tissue damage are among the most important treatment components. The use of low-air-loss or air-fluidized beds is generally indicated for stage 3 and 4 pressure ulcers.^4^,^14^ Pressure mapping can help identify focal areas of pressure threat while aiding in the design of reductive strategies. A patient at risk for an ischial pressure ulcer development should avoid prolonged sitting and use pressure-relieving seat cushion. Identifying nutritional deficiencies and achieving a positive nitrogen balance cannot be overemphasized.^4^,^14^

## LOCAL WOUND MANAGEMENT PRINCIPLES

Regardless of the specific wound type, general local wound management principles exist for a wide variety of chronic wounds.^4^ The TIME acronym, promoted by the Wound Healing Society, is a simple, yet comprehensive method for defining, communicating, and addressing principal elements associated with impaired wound healing.^15^ The letter “T” refers to tissue, dentifying specific tissue deficits as well as the presence of devitalized or necrotic tissue. The letter “I” characterizes inflammation or infection within and surrounding the wound site. The letter “M” reflects the state of moisture balance, ranging from maceration to desiccation. The letter “E” describes the quality of the wound edge, often heaped up, nonadvancing, and hyperkeratotic in the chronic wound setting, while also describing the extent of reepithelialization.^15^

## TISSUE

The initial step in the management of any chronic wound is to remove the local impediments to wound healing by eliminating devascularized tissue, necrotic material, and excessive bacterial burden.^15^ Modern wound bed preparation strategies involve a thorough and yet judicious debridement, preserving vital tissue while ridding the wound site of the accumulated impediments to optimal healing. In so doing, we convert the poorly healing or impeded chronic wound state to one resembling an acute wound.^15^^,^^16^ At present, numerous modalities are available for debriding wounds. They include the use of sharp surgical instruments (scalpel), mechanical devices like curettage and waterjet, enzymatic agents like collagenase and papain-urea derivatives, autolytic debridement dressings like hydrocolloid and occlusive dressings as well as biological interventions including the use of maggots.^15^–^17^ At this time, no definitive evidence establishes any single form of debridement as superior in reducing healing time. Sharp debridement is generally regarded as fast and effective particularly in cases of pressure, diabetic, and venous related ulceration.^16^ In cases of significant arterial insufficiency, radical debridement should be performed after revascularization, unless complicated by sepsis.^5^

## INFECTION

In a wide variety of wound types, uncontrolled and self-sustaining inflammatory mechanisms are considered responsible for the failure of chronic wounds to heal.^18^ Decreasing bioburden to subinfection levels facilitates control of local and systemic inflammatory mediators. Quantitative tissue biopsies and validated semiquantitative swab techniques provide objective evidence of control of the bacterial burden and help to qualify and speciate the offending pathogen. Bacterial concentrations exceeding 10^5^ or 10^6^ bacteria colony-forming units per gram of tissue, or any level of β-hemolytic streptococci, have been shown to impair wound healing and surgical closure.^5^ In both the United States and Europe, *Staphylococcus aureus* continues to be the most commonly identified pathogen in chronic wounds, with methicillin-resistant *Staphylococcus aureus* accounting for upward of 20% to 50% of cases. This is true for both inpatient services as well as dedicated wound care centres. As a result, a resistogram is essential to guide appropriate therapy.^19^ Surgical debridement and topical antibiotics effectively lower the number of bacteria in chronic wounds.^17^,^20^ Systemically administered antibiotics do not effectively decrease bacterial levels in granulating wounds, whereas topically applied antimicrobials can be effective.^20^ The use of silver-containing dressings has increased significantly over the past years with multiple reports relating improved rates of healing. To date, three randomized control trials have not demonstrated a significant increase in complete ulcer healing.^21^

Systemic infection, acute foot infections, and local cellulites should be treated by systemic antibiotics.^5^ Once in bacterial balance, the use of topical antibiotics should be discontinued, as protracted courses of antibiotics may inhibit wound healing and promote the development of resistant organisms.^21^ Osteomyelitis, best confirmed by bone biopsy, requires systemic antibiotics, vascularized soft tissue coverage when lacking, and possible surgical intervention.^5^

## MOISTURE

General wound cleansing should be performed using nonirritating and nontoxic solutions to minimize additional trauma and cytotoxicity. Many current dressings combine components of wound bed preparation, that is, debridement and antimicrobial activity, with moisture control. Maintenance of a moist (not macerated) environment is accepted as the best topical environment for open wounds.^13^,^22^

Choosing an appropriate wound dressing should consider the current phase of wound healing, its specific temporal requirements, as well as potential side effects. Ideally, dressings should minimize pain and be easy to use. These dressings must prevent friction and shear while protecting the peri-ulcer tissue and skin.^13^ A review of the current literature advocates the use of hydrogels for the debridement phase, foam at the granulation stage, and the use of either hydrocolloids or low adherence dressings for the epithelialization phase.^13^ Interestingly, a recent review noted that a single modality therapy consisting of either a paraffin gauze dressing or a saline-moistened dressing can also be effectively employed.^22^ With perhaps the exception of hydrocolloid dressings, at present there is little concrete evidence to prove superiority of modern dressings in terms of general performance criteria (ease of use, pain, ability to absorb and contain exudates, avoidance of wound trauma on removal).^22^ There is an emerging evidence that negative pressure therapy, applied after debridement, is helpful in decreasing local oedema, removing fluid and local debris, increasing peripheral wound perfusion, promoting granulation tissue formation, and decreasing overall wound size in both animal models and in cases of lower extremity ulcers.^23^,^24^

## EDGE OF THE WOUND

Wound healing progress and general wound conditions should be monitored regularly; ideally, by the same caregiver each time. An appropriate therapeutic response should demonstrate a reduction in ulcer size; if not, a biopsy should be taken to rule out other underlying diseases like squamous cell carcinoma and pyoderma grangraenosum. Drug-related and systemic autoimmune diseases should also be considered.^5^

Surgical procedures can be divided in those that provide definitive closure of the wound and those that treat the underlying disease. Nutritional status, bacterial load, hemodynamic considerations, and vascular status all play extremely important roles in the timing of definitive surgical repair.

When faced with exposed functional elements like tendons, bone, or neurovascular structures, prompt surgical intervention for protection and preservation is of particular urgency. It is interesting to note that even when skin grafts are used for the treatment of venous ulcers, no definitive evidence exists demonstrating that either long-term or overall healing times can be shortened.^5^ When severe lipodermatosclerosis complicates a chronic wound, free flap reconstruction after thorough excision, debridement, and bacterial control has been shown to accelerate healing.^25^ Surgical closure of pressure ulcers is generally recommended only if, despite all efforts at prevention and optimization, the wound fails to heal in a timely fashion.^4^,^25^ Composite tissue closure affords the best chance of sustained wound closure. Temporary fecal or urinary diversion may occasionally be required to facilitate wound healing.^5^ It is important to note, however, that closing wounds by the aforementioned procedures, without paying attention to the underlying disease, is not a long-term solution and is prone to recurrence.^5^

## ADJUVANT AGENTS

A wide variety of commercially available adjuvants are marketed to facilitate the treatment of chronic wounds. Unfortunately, quality, randomized controlled trials continue to lag behind promotion and application. Improved functional status, ABI, and quality of life have been documented with the use of cilostazol when treating arterial ulcers.^26^ Pentoxifylline^27^ and the application of bilayered artificial skin dressings,^5^ both utilized in conjunction with elasteric multilayer high-compression bandaging for the treatment of venous ulcers, have been validated as has the application of platelet-derived growth factors for neuropathic ulcers^28^ and pressure ulcers^5^ therapy. Recent concerns regarding malignancy with the use of Regranex have raised specific concern regarding its use.^29^ Electrical stimulation, ultrasound, low energy laser, spinal cord stimulation, and the application of hyperbaric oxygen therapy are promising therapies with theoretical, rational, and preclinical studies suggesting their use.^5^ Quality randomized controlled trials concerning their efficacy in chronic wound care are at present lacking. Negative pressure wound therapy has shown some evidence as an adjunct for healing challenging wounds.^5^ Laser therapy and phototherapy have not been shown statistically to improve ulcer healing.^5^

## ULCER RECURRENCE

Reported recurrence rates for most chronic ulcer types remain extremely high, ranging from 23% to 40% for pressure ulcers, 24% to 57% for venous ulcers, and upward of 60% for diabetic ulcers supporting the importance of preventive efforts.^14^,^30^,^31^ Primary diagnosis and treatment, identification of risk factors, management of comorbidities as well as directed attention to risk factors and education remain key to successful prevention of recurrence.^5^,^14^,^30^,^31^ Antiplatelet therapy and the reduction of risk factors like smoking cessation as well as control of diabetes, hypertension, hyperlipidemia, and elevated homocysteine levels are specifically advocated for arterial ulcers.^5^,^30^,^32^ Exercise has proven to be beneficial for both arterial and venous ulcer prevention, and consistent use of compression therapy and surgical correction of superficial venous reflux has been shown to be essential for prevention and healing of venous ulcers.^5^,^12^,^30^,^33^ The use of protective footwear and, most importantly, meticulous attention to foot care including proper bathing and nail trimming have been shown to reduce the incidence and complications associated with diabetic foot ulcers.^5^,^8^,^34^ Notable reductions in the incidence of pressure sore development of up to 60% have been demonstrated with the use of pressure-reducing strategies, as well as the utilization of appropriate surface and bed types.^5^,^35^

The importance of nutritional assessment and optimization cannot be overemphasized in the management of patients with chronic wounds. Malnutrition is prevalent in many of our elderly patients and commonly manifests in patients with chronic and systemic diseases, gastrointestinal disorders, malignancies, traumas, immunosuppressive states, and associated drug therapies among others. Several different tools have been developed to screen for undernutrition. Two commonly employed tools are the Birmingham Nutrition Risk score and the more recent Malnutrition Universal Screening Tool. This latter score was developed by the Malnutrition Advisory Group of the British Association of Parenteral and Enteral Nutrition (BAPEN) for use in all healthcare settings. Malnutrition Universal Screening Tool has been validated against a number of established modalities, demonstrating excellent reproducibility, good to excellent agreement with the Birmingham Nutrition Risk and acceptable in practice to both patients and healthcare workers. We have found this tool to be particularly beneficial for routine screening of nutritional status in patients with chronic wounds and recommend it accordingly.^36^

One of the most important and often-neglected aspects of wound management is the proper education of patients and family members. Patient education has been shown to improve the quality, frequency and efficacy of dressing changes, compliance as well as the treatment and prevention of recurrence.^5^,^37^ Optimal remunerative strategies and the implementation of support mechanisms as well as the development of an efficient infrastructure are at present severely lacking, further complicating the situation.

Encouraging and updating medical staff and caretaker education, while critical for success, remain an often-neglected role in healing and preventing chronic wounds.^38^ At present, no evidence-based method exists to educate nurses, general clinicians, or practitioners. We and several authors advocate formally devoting a portion of the core educational curriculum of medical students, nurses as well as general clinicians and healthcare providers of tomorrow to the importance and understanding of wound pathophysiology and treatment. The quality of which should be reinforced and substantiated by practical teaching (eg, skill labs, bedside-teaching) and examination (eg, OSCE, Mini-CEX).^38^–^40^

## FUTURE

Emerging technologies present novel approaches to future wound care. In the near future, gene therapy may allow genes or gene-derived messengers important in healing to be delivered directly into a wound at directed time points.^41^ Skin and composite equivalents from embryonic stem cells and application of bone marrow–derived stem cells seem further possible options.^42^ These future developments will depend very much on public and professional support for further research.

Unfortunately, one of the major barriers to effective wound care continues to be the lack of interest, enthusiasm, and knowledge shown by many clinicians and general practitioners for this subject. To improve chronic wound care in the near future, there must be changes made in the medical student curricula to increase wound education and awareness at all levels. Wound-related education leads to improved communication, continuity of care, shortened hospital stays, and reduced costs and will help further accelerated progress of chronic wound care in the future.
